# Development of an Integrated Suite of Software in Analysing of Large DNA Databases

**DOI:** 10.2174/1874431100802010001

**Published:** 2008-02-13

**Authors:** K.S Kong, E.Y.K Ng

**Affiliations:** 1 *DS Center/IEP, DSO National Laboratories, 20 Science Park Drive, Singapore 118230*; 2 *School of Mechanical & Aerospace Engineering, Nanyang Technological University, 50 Nanyang Avenue, Singapore 639798*

## Abstract

The work showed that the integrated suite of software tools for detecting criminals using DNA databases has achieved the overall objective by providing a working platform for sequence analysis. The work also demonstrated that by integrating BLAST and FASTA (two widely used and freely available algorithms), plus an additional implementation of PSA (custom-built pairwise sequence alignment algorithms) and TR analysis tools (for detecting tandem repeats) with the rest of the utilities supporting tools (databases and files management) developed, it is entirely possible to have an initial working version of the software tool for criminal DNA analysis and detection work. The integrated software tool has great potential and that the results obtained during the tests were satisfactory. The recent South Asia Tsunami incident has renewed the need to establish a quick and reliable system for DNA matching and comparison. This work may also contribute towards the quick identification of victims in many disasters.

Future works are to further enhance the existing tools by adding more options and controls, improve upon the visualisation display, and to build robust software architecture to better manage the system loadings. Fault tolerance enhancement to the system is one of the key areas that can further help to make the entire application efficient, robust and reliable.

## 1 INTRODUCTION

A murder has been committed. Apart from the victim, there are no other witnesses, but some evidence has been found at the crime scene. Blood and hairs were gathered by the crime scene investigators, and it was believed that it could be left by the suspect. From the evidence collected, would they be sufficient to provide a clue as to how the murder was committed and who was the murderer? Imagine a deoxyribonucleic acid (DNA) profile (i.e. double helix DNA typing [1-4] can be constructed from those physical evidences found at the crime scene and used to match against a DNA database of known suspects. By using a computer DNA analysis tool to query a large human DNA database, the result obtained from the analysis would be the evidence that can be used to either convict or acquit a suspect.

DNA typing applications are not only computational intensive and time consuming, the cost of implementation to develop such a computer system is also very expensive and technological demanding. However, until recently, the costs of hardware and software have been reduced significantly, making it possible now to design and build high performance computerized systems to carry out DNA analysis on large DNA databases to assist in the area of forensic science to detect criminals. In addition to these issues relating to technologies, there are also issues related to the accuracy of the DNA fingerprinting analysis. The two major concerns are that (1) a DNA fingerprint belonging to a specific person must have a high probability. In other words, the confidence level that a correct match is obtained must fall within an acceptable range, and (2) the DNA sample for analysis must not be contaminated by any external sources, therefore it is essential and important to ensure the integrity of the DNA sample. The result of the DNA analysis will be detrimental if the standard process is not being followed closely. Due to the importance of such systems, even a slight error can have a profound impact on the outcome.

The aim of this study is to develop an integrated software tool, using the DNA typing concept and genetic sequence analysis algorithms (i.e. sequences alignment technique) for detecting criminals using DNA databases. The initial phase of the study is to understand the requirements of building a DNA database system that can assist forensic experts to carry out an automated search and match operations on a DNA database. Once a general understanding is achieved, the next step is to design and build a software system that will fulfil such needs. A number of important sequence alignment algorithms will be reviewed in section 2 and chosen for the implementation of this software system. The end delivery of this study is a working DNA analysis tool, a cost effective one, which serves as a proof of concept application and will work on test data stored in the DNA databases.

The DNA analysis application will be built using Netbeans Integrated Development Environment (IDE). Netbeans IDE (current version is 3.6) is an open source WYSIWYG Java development tool (current Java version is 1.4.2). The application is designed and developed to run on Microsoft Windows XP. Since the application (which includes graphical display and sequence analysis algorithms implementation) is written using Java programming language (version 1.4.2), it can be run on any computer platforms which has a Java Virtual Machine (JVM) installed. The database schema will conform to the FASTA format (i.e. a format that is commonly used for sequence search) and stored using facilities provided by *formatdb* and *Biojava*.

The system architecture of the application has a database to contain all the criminal DNA data, a set of DNA sequence analysis algorithms, and a Java GUI based DNA analysis tool for results display and sequences manipulation facilities. However, due to the confidentiality and sensitivity nature of the data (i.e. involving human DNA), a high security clearance from the relevant authorities is required in order to have access to the real data, which makes it difficult to acquire real data for the testing. Therefore, in this work test data, a combination of self-generation data supplemented by data obtained from the Internet Genome Databases will be used. Nevertheless, it is sufficient for testing the software application and for the purpose of concept demonstration.

Since the subject matter on DNA typing is wide, and with only limited resources and time, the emphasis of this study is therefore confined to the implementation of sequence alignment algorithms, such as pairwise sequence alignment (PSA), short tandem repeats search algorithm, BLAST and FASTA alignment algorithm, in the area of forensic science applications.

## 2 LITERATURE REVIEW

This section provides an overview of the concept of DNA Typing and its scientific basis. It also provides a detailed study into various DNA sequence alignment algorithms that are hitherto available.

### 2.1 The Scientific Basis of DNA Typing

Often, DNA fingerprinting deals with DNA sequences at the molecular level. Therefore, it is important to understand terminologies like locus, allele, polymorphism (and their different types) and tandem repeats. These are discussed in the following paragraphs.

The physical location (i.e. at the molecular level) in the genome is called locus (singular – locus, plural – loci). The presence of multiple alleles (i.e. alternative forms of a single gene) of a marker at a single locus is known as polymorphism. There are two kinds of variation (1) sequence polymorphisms and (2) length polymorphisms. Sequence polymorphisms are usually simple replacement of one or two bases in the genes themselves [5]. For example, Fig. (**1**) depicts the sequence polymorphism at the fifth base pair from the left.

This is different in the case of length polymorphisms. Length polymorphisms are known as the variations in the length of the DNA molecule. For example, given three blocks of repeats (with similar DNA sequences), each block is known as a tandem repeat as shown in Fig. (**2**).

Therefore, a locus that exhibits variation in the number of tandem repeats is known as a variable number tandem repeat (VNTR).

Research has shown that only 5% of the human genome contains useful genes. Genes are portions (sub-units) of chromosome that contain useful information and serve as templates for the production of proteins. However, the rest of the 95% of the human genome does not contain genes that code for any proteins. Sometimes this section of the human genome is known to be “junk DNA”. Until recently, studies have shown that these “junk DNA” do have important functions such as regulating gene expression, assisting in cellular machinery and serving as chromosomal structure support [3].

It is in these non-coding regions of the human genome where the VNTRs are mostly located. The number of copies of a VNTR determines the size of a DNA fragment, and each individual has a unique number of tandem repeats at specific molecular location (i.e. loci) on his or her chromosome. Essentially, this important principle serves as a building block of DNA evidence that is used today in many forensic works to allow unambiguous identification of suspects.

### 2.2 The Scientific Basis of DNA Fingerprinting

After gaining understanding of the scientific basis of DNA typing, we now turn to a few sequence alignment algorithms that perform appropriate tasks. In many applications of Bioinformatics, especially in the area of forensic studies, we need to examine the possible optimal alignment between two or more related sequences (i.e. be it DNA or protein sequences, these sequences can be extracted from various loci with tandem repeats or from any forensic evidences) and the close relationship between them. For this reason, it is important to include them in our studies and understand their vital roles and functions in forensic applications.

### 2.3 Dot Plot

One of the simplest methods for evaluating whether two sequences are similar is to use a dot plot approach [6]-[7]. First, a matrix is constructed, with sequence A on the x-axis and sequence B on the y-axis. Begin with all the cells initialised at zero. The computation starts by taking the first character of sequence A and comparing it with all the characters of sequence B. Mark those characters of sequence A with an ‘**X**’ if the same character can be found in sequence B. Next, the computation advances to the next character of sequence A and the same steps are performed. Stop when all the characters in sequence A have been compared with all the characters of sequence B. At the end of the computation, a dot plot is produced as illustrated in Fig. (**3**).

One of the shortcomings of a dot plot is that it can be complex and overcrowded with dots when the sequences become too large and similar (i.e. similar does not mean identical). Normally, identical sequences will give rise to a single diagonal line across the plot as shown in Fig. (**4**). Whereas in Fig. (**5**), similar sequences tend to produce a broken diagonal line and the gaps in between them indicates that there are mismatches between the two sequences. If the two sequences are distantly related with very few similarities, it will not only contain more diagonal lines in the direction parallel to central diagonal, but also broken diagonal lines as in Fig. (**6**). The distance between the central diagonal line and the surrounding sub-diagonal lines represents the correction needed by introducing gaps to align the two sequences.

Since the dot plot is particularly sensitive to noise when comparing two large sequences with similarities, one of the workaround solutions is to introduce the concept of a sliding window with a cutoff threshold. The sliding window is similar to a cart that contains a number of characters (i.e. defined by the window size), which will be used and compared with other characters in another cart. If the total number of matches between these two carts is higher than or equal to the predefined cutoff threshold, a match is found, otherwise no match is found and the sliding window then advances to the next set of characters and the process is repeated. The sliding window with a cutoff threshold helps to reduce noise in the dot plot.

### 2.4 What is a Pairwise Sequence Alignment?

An alignment between two sequences is defined as a pairwise match between the characters of each sequence. So, pairwise sequence alignment is a technique used to find the optimal pairing of sequences (i.e. can be either DNA sequences {A, C, G, T} or Protein sequence {A, C, D, E, F, G, H, I, K, L, M, N, P, Q, R, S, T, V, W, Y}) that preserves the order of characters in each sequence. Gaps may be introduced in the alignment process so that the total score can be maximized [6-9]. This concept can be generalised as follows in Table **1**.

With the generalisation and understanding of pairwise sequence alignment, we are now ready to tackle more sophisticated algorithms. The algorithms described in the following sections perform sequence alignment *via *dynamic programming. Dynamic programming is a concept, which refers to solving an instance of a problem by taking advantage of computed solutions for smaller subparts of the problem. Three alignment methods will be reviewed in the following sections, namely global, semi-global and liner alignment algorithms. In addition, two penalty schemes, the linear and affine gap penalties, will be discussed for each alignment method.

#### 2.4.1 Global Alignment With Linear Gap Penalty

Regardless of the location of the gaps in a sequence, global alignment will give penalty to gaps identified during sequence comparison. As such, the entire sequence will be considered as a whole entity during the alignment process. The “Needleman and Wunsch Algorithm” is one such global sequence alignment algorithm. The algorithm is described in Table **2**.

#### 2.4.2 Semi-Global Alignment With Linear Gap Penalty

If the aim is to use a shorter sequence to search for a larger sequence for a possible sub-sequence match, semiglobal alignment will be a better choice as compared to the global alignment as the latter will penalise gaps at either ends of the alignment. Semi-global alignment solves this problem by not penalising gaps found at either ends of a sequence. The semi-global alignment with linear gap penalty is described in Table **3**.

#### 2.4.3 Local Alignment With Linear Gap Penalty

If the aim is to find sub-sequences that are similar to any part of a long sequence, both the global and semi-global alignments are not suitable because both penalise every non-matching position. Hence, the local alignment is proposed to solve this problem and one such method is the “Smith-Waterman Algorithm”, which is described in Table **4**.

#### 2.4.4 Global Alignment With Affine Gap Penalty

In many occasions, gap of length *k* is more probable than *k* gaps of length 1. This is especially so for a single mutation event that can insert or delete a stretch of characters in a sequence. In addition, distinct mutational events could also lead to separated gaps being produced. A linear gap penalty function treats all these events in the same fashion. However, the affine case distinguishes these events and treats them separately. The affine gap penalty function uses two penalties, which are the gap opening penalty, referred to as *h*, and the gap extension penalty referred to as *g*. The only difference between a global alignment with linear gap penalty and a global alignment with affine gap penalty is the penalty function being used, and instead of only one similarity matrix being computed (in linear gap penalty), three similarity matrices are being computed (in the affine gap penalty). The rest are similar. The global alignment with affine gap penalty is tabulated in Table **5**.

#### 2.4.5 Semi-Global Alignment With Affine Gap Penalty

Table **6** summarised the semi-global alignment with affine gap penalty.

#### 2.4.6 Local Alignment With Affine Gap Penalty

The local alignment with affine gap penalty is detailed in Table **7**.

#### 2.4.7 Comparison of Global, Semi-Global and Local Alignment

Krane *et al*. [6] reviewed that global alignment is good for comparing two sequences in their entirety, if this is the intention. The gap penalty is scored regardless of where the gaps are located; the gaps can be found in the middle of a sequence, or at either end of a sequence. However, to locate a sub-sequence within a longer sequence (but not the entirety), semi-global alignment is more suitable. This is because semi-global alignment does not penalize gaps that are located at either end of a sequence. Quite often, gaps that are located at either end of a sequence have no biological significance and therefore, it can be safely omitted without any significant impact. But gaps that are located within a sequence will be penalized. There may be times where only a very small sub-sequence matches a subsection of a larger sequence, and there are many mismatching position, the alignment score may be lowered significantly. Under such a circumstance, it is better to use local alignment. Local alignment ignores mismatches and gaps before and after the matching region, but it reveals the matching region in the centre of two sequences.

Although each PSA algorithm has its own strengths and limitations, it is able to meet most of the sequence alignment needs. In fact, PSA works with thousands, or even millions, of DNA and protein sequences, which would otherwise be impossible if done manually.

## 3 HEURISTIC ALIGNMENT ALGORITHMS

The above-mentioned PSA algorithms work efficiently when aligning a smaller set of sequences. However, it is more common to perform pairwise database search using a query sequence through a database of many sequences to retrieve those that are similar to that query sequence. This can quickly translate into a higher demand in the usage of computing resources, such as hardware memory, disk space and CPU speed, which is not a trivial task. Therefore, two important techniques were developed to handle these requirements. They are BLAST [10] and FASTA [11] database search techniques, which are simply extensions of the PSA technique and are fast because they incorporate heuristic features in the algorithm. In the following sections, we will look at these well-known techniques in greater detail.

### 3.1 BLAST

BLAST (Basic Local Alignment Search Tool) algorithm was originally developed in 1990 by Altschul *et al.* and it is one of the most commonly used tools for searching sequence databases for maximal un-gapped local alignments. It is efficient and optimised for parallel computation. BLAST adopts a simple approach by taking the input sequence and breaking it down into a fixed length of words (normally length of 4). After which, these words will be used to search through the sequence databases to obtain high-scoring pairs. The BLAST search process can be summarised as follows:

Given an input sequence: ACCGTTTAAAA

Step 1:	Break the query sequence into words of a fixed length (default word length of 4).

ACCGTTTAAAA **→** ACCG, CCGT, CGTT, GTTT, …, AAAA

Step 2:	Pre-process the words by discarding those that contain common amino acids.

Step 3:	Starting from “ACCG”, search the sequence databases for word matches.


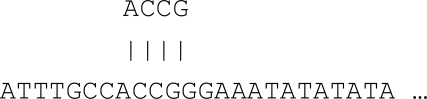


Step 4:	Then, extend the query sequence and repeat the search until the local alignment score falls below a certain threshold.


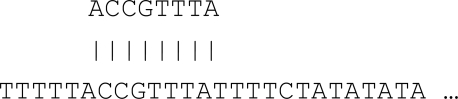


Step 5:	Output the alignment results.

There has been much work done lately on improving sequence alignment such as Clustal W is the most commonly used multiple alignment software [12]. ClustalW can generate phylogenetic trees when a properly formatted alignment is input.

### 3.2 FASTA

FASTA, originally developed in 1985 by Lipman *et al.* [11], is used to perform gapped local alignments between sequences. Similar to the BLAST technique, FASTA also breaks a query sequence into words of a fixed length, known as ktup no. For nucleotides, ktup no 4 to 6 is used, and for proteins, ktup no 1 to 2 is used. Next, a look-up table and an offset table are constructed for a query sequence and a target sequence respectively. By comparing these two tables, similar subsequences are identified. The FASTA search process can be summarised as follows:

Given a query sequence: ATGCTATAC


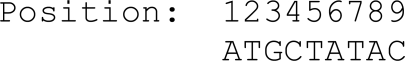


For a ktup no = 4, a look-up table is constructed as follows:

**Table:** 

Word	Position
ATAC	6
ATGC	1
CTAT	4
GCTA	3
TATA	5
TGCT	2

Given a target sequence: TGCTAT


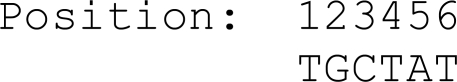


The following offset table is constructed using ktup no = 4.

**Table:** 

Word	TGCT	GCTA	CTAT
Position	1	2	3
Offset	1	1	1

Offset by 1 (a large no. of instances of the distance 1 in the second table), we would obtain the following alignment.


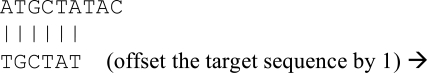


### 3.3 Comparison Between BLAST and FASTA

The similarities and differences between the BLAST and FASTA approaches can be summarised as follows [13]. BLAST and FASTA have the following common strategies:

- Both techniques have fast screening process to eliminate unrelated sequences; and
- Both techniques are able to complete alignment of top scoring sequences.

The differences between these two techniques can be found in:

- Statistical model; and
- Heuristic and tuning.

## 4 TANDEM REPEATS SEARCH ALGORITHMS

In section 2, the definition of tandem repeats and its application were introduced. A brief discussion for each of the tandem repeats alignment algorithm is given here.

Tandem repeats (TRs) can be generally classified into two categories. The first being the exact tandem repeats (ETRs), which refer to repeats that exist in two or more duplications, and each repeat following the preceding one in a continuous fashion. The second category is the approximate tandem repeats (ATRs), which refer to repeats that evolve through mutations (i.e. insertions, deletions or substitution), and each repeat following the preceding one may differ slightly in one or more alphabet. For example, …AGCTAGCTAGCT… is an ETR with “AGCT” repeated three times in a continuous manner and …AGCTAGTTAGCTAGCCAGCA… is an ATR with AGCT**→**AGTT**→**AGCT**→**AGCC… Take note of the variation in one of the alphabets. The remaining sections give an overview of TR algorithms being used currently.

In a paper written by Benson [14], he grouped the current TR algorithms into four general approaches. These are:

- Alignment matrix approach. This approach computes and aligns alignment matrices and adjacent repeats within a DNA sequence. However, the limitation of this approach is excessive running time.
- Data compression approach, which takes advantage of the fact that adjacent repeats can be compressed efficiently into respective regions and that these regions contain tandem repeats.
- Heuristic approach. This is a direct approach, whereby it incorporates heuristic techniques during implementation. However, not all tandem repeats can be identified through this approach.
- Direct approach. This approach, unlike the previous three approaches, aims to search for tandem repeats directly. A Tandem Repeat Finder [14] is an example of such a method.

Due to the complexity and wide variation of each TR algorithm, it is not possible to discuss every one. However, to complete the discussion, two methods have been selected for a walkthrough, so that the steps taken to search for tandem repeats within a nucleotide sequence can be better understood.

## 5 ARCHITECTURE DESIGN AND COMPONENTS IMPLEMENTATION

In this section, a detailed discussion is provided in the area of architecture design and implementation.

### 5.1 System Architecture Overview

Fig. (**7**) depicts the high-level system architecture of the system developed. The user interacts with the system *via *a GUI (Graphical User Interface) display to perform sequence alignment operations. The sequence alignment algorithms included in this work are PSA, BLAST, FASTA and Dotplot. In addition, TR is used for tandem repeats analysis. PSA, TR and Dotplot are custom-built applications using the Java SDK, whereas BLAST and FASTA are both open-source applications freely available for development use and have been integrated to the system. The rest are utilities developed to support Sequence Analysis Work. Examples are a set of database utilities for index (key) creation, data conversion, and packaging of data into the Fasta data format (i.e. BlastFastaData) for database search, which are particularly used by BLAST and FASTA algorithms. Also, BioJava utilities for creating and maintaining data (i.e. BioData) that is compatible to BioJava standard, are used by the PSA algorithm. BioJava also provides many other sequence routines, algorithms and libraries for building bio-applications. In addition, a text file editor is also developed to facilitate sequence files editing.

PSA, BLAST and FASTA algorithms are capable of utilising multiple CPUs for parallel execution. PSA achieves this *via *the Java Threading function. Java Threads are light-weight processes spawned by PSA to perform database search and sequence alignment. Open-source such as BLAST and FASTA were developed to handle multiple CPUs as well.

### 5.2 Components Implementation

	The GUIMain is the root user MMI Interface. It was built using the Java Frame Class. The rest of the user interfaces (such as the Dotplot MMI and PSA MMI) are to reside within this root MMI as Java Internal Frame Class. In other words, the GUIMain is the main console where everything begins.

The Dotplot software component has four input parameters as shown in Fig. (**8**). They are the input sequences to be compared and their homology determined *via *the Dotplot algorithm. The window size determines the word size on each axis to be examined, moving from left to right for the horizontal axis (sequence A) and moving from bottom to top for the vertical axis (Sequence B). A dot will be plotted on (x, y) coordinate if the number of matches between sequence A[x, x + windowsize] and sequence B[y, y + windowsize] > threshold on a diagram. The output is a plot diagram indicating those sections with two identical sequences as illustrated in Fig. (**9**).

A GUI was developed using the Java Internal Frame (Java Swing Library) and integrated with the Dotplot algorithm. The user specifies the input parameters, such as sequences to be compared, the window size and the threshold, and the type of file to be imported (i.e. either DNA or protein sequence file) and click on the <Apply> button to run the Dotplot program. The result of the plot is displayed on the “Results” panel as presented in Fig. (**10**).

**Fig. (11):**
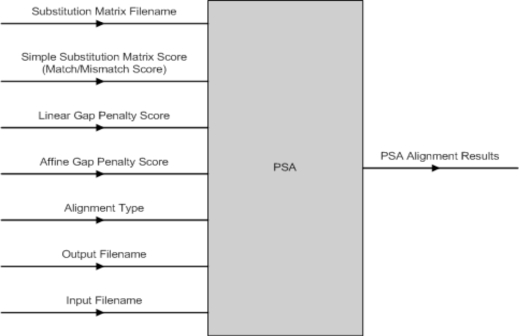
PSA Input & Output Parameters.

The PSA software component has a number of input parameters that can be set (Fig. **11**). The PSA component was implemented to perform three different types of alignment with either linear or affine gap penalty function depending on the user’s selection. They are (1) Global Alignment with linear or affine gap penalty, (2) Semi-Global Alignment with linear or affine gap penalty and (3) Local Alignment with linear or affine gap penalty.

The PSA GUI was implemented as a Java Internal Frame. It has controls to handle all the three types of PSA discussed earlier on. That is, the user is able to select a single query sequence to search against a database containing many target sequences, or to search against a file containing multiple target sequences, or to search only one target sequence. Once the user has selected the desired option, he/she will be prompted to enter the desired values. Finally, to execute the application, the user can click on the <OK> button to run the PSA operation. The result is displayed on the “Results” text area, as seen in Fig. (**12**).

For the BLAST component, only the essential parameters are implemented. This is sufficient to demonstrate that the BLAST Algorithm can be integrated into the GUI and can work together as one single component for the present application. The remaining parameters can be incorporated into the existing architecture easily.

The GUI was developed from ground up. The layout was designed to accommodate a partial list of BLAST input parameters (not the full parameter list). Hitherto, the remaining input parameters are left out. However, the GUI was developed to allow future expansion in mind without too much hassle. If needed, the GUI can be extended using the Netbeans IDE GUI Editor Tool. The BLAST GUI is shown in Fig. (**13**).

To input parameters, sequences file and target database, the user clicks on the “folder” icon. To run the BLAST application, the <OK>  button is clicked. The alignment result is shown on the result text area.

FASTA, like BLAST, is also a freely available open-source application that can be downloaded from the net. Hence, it is not required to build from ground up. FASTA is incorporated together with the GUI, which is developed from scratch. Likewise, only a partial list of parameters are used, which is sufficient for concept proofing. If needed, the remaining parameters can be incorporated for future work.

The FASTA GUI is implemented and it has controls for files input and parameter settings. To execute the FASTA alignment, click on the <OK> button. The result is then displayed on the text area.

DNA fingerprinting is the foundation of most forensic applications. The uniqueness of DNA fingerprinting is based on the number of tandem repeats found in each individual “junk DNA”. It is this tandem repeats that give rise to unique identification of an individual. Therefore, TR analysis tool is included in this software implementation.

Due to the high complexity and difficulty of ATR implementation, the TR component in this implementation only handles detection of ETR as this is sufficient to demonstrate the concept of using TR Search Tool to identify potential criminals. The inclusion of ATR implementation can be considered for future work.

The inputs to TR search tool are: (1) suspect’s DNA sequence; and (2) minimum and maximum TR word size. During stage 1, the suspect’s DNA sequence is analysed and the TR tool will locate all possible ETRs found within the nucleotide sequence based on the selected TR word size. The tool allows the TR word size to be configured freely, as well as to shorten or widen the sliding window size (the sliding window is used to scan along the sequence and to locate possible TRs). Once all the possible TRs are identified, the user is able to select one or more TRs to scan the selected databases for a possible match. If one or more matches are found, the results are displayed on the MMI. See Fig. (**14**).

Fig. (**15**) depicts the GUI layout of the tandem repeats search tool. To upload a suspect’s DNA sequence, click on the “folder” button. A file selector dialog will be prompted. Enter a minimum and maximum word size for the TR search. Click on the “Enter” button to start analysing the DNA sequence. The TR algorithm will perform TR analysis on the sequence being entered. All the possible ETRs are displayed in the STR Word Text Area. For instance, “ATG (3)” means that “ATG” is found to be a continuous TR within the sequence and “(3)” means that there are three such TRs.

The next step is to select one or more STR words to scan through one or more databases (multiple selection of databases are allowed). Click <OK> to execute the database scan and the final results of the suspect’s ID and the associated DNA sequence in the databases.

As Bioinformatics advances, there is an ever-increasing need to store large amounts of biological data for analysis work. It is common to work with very large sequence databases that are unable to fit into the current physical memory available. Hence, the preferred way to handle large amounts of biological sequence is to use a dedicated database system. FASTA and BLAST provides a “Fasta” format database for this purpose. They use the “formatdb” routine to create their databases. BioJava also provides a set of library APIs (Application Programming Interfaces) for data creation, update and modification.

As such, a number of database tools have been developed in this work for such purposes and are summarised as follows:

1. Tool to create “Fasta” format database:
  - The “Fasta” format is widely accepted by FASTA and BLAST applications. In fact, it is one of the most widely used data format in many of the third-party applications.
  - In this work, both of the FASTA and BLAST use this format.
2. Tool to create BioJava compatible format database:
  - BioJava offers a simple and efficient sequence database implementation backed by one or more sequence files on disk. These files can be in any format, as long as a suitable sequence format class exists.
  - The current PSA software component uses this format.
3. Text File Editor:
  - To create, modify and update sequence or database files.

To create a local customised database for BLAST and FASTA applications, the “formatdb” routine, which is provided by the BLAST and FASTA packages, is used. However, it is rather inconvenient to use the “formatdb” command manually *via *the command line input. Therefore, a GUI is developed to provide a more user-friendly interaction.

To upload a file containing the sequences, click on the “folder” icon. After that, select the appropriate file type. Choose either “NUCLEOTIDE” for DNA sequences or “PROTEIN” for protein sequences. By default, it is set to “FALSE” for parse output. Click the <OK> button when done to initiate database creation.

For the PSA component, the BioJava compatible format database is used. However, BioJava Library only provides a list of APIs, and hence there is a need to build these database tools.

The first database tool is the “Create DB Index” routine. This routine is responsible for creating a BioJava DB Index for storing all the sequences on disk. It functions like a schema. Fig. (**16**) depicts the GUI layout.

The second database tool is the “Add File To DB”. This routine is responsible for uploading all the sequences to the DB Index created earlier on. Only when this step has been executed, then can the DB be populated with data.

The last database tool is for listing all the sequence IDs associated with a particular database. It is useful when a global listing of sequence IDs is required.

The sequence file-editing tool is responsible for creating; updating and modifying sequence files or sequence databases. These files can then be uploaded *via *the appropriate database tools into various data formats, which can be used, by BLAST, FASTA and PSA.

The data used for testing the software tools is generated from a custom-built software program. Fig. (**17**) shows the program has four parameters, which are:

1. Record Size - the number of records to be created;
2. Repeat Word - the TR pattern to be inserted into the test data;
3. Random Seed - to add in random variation; and
4. Record Name - the record name for each sequence generated. It is postfix by a unique ID generated by the computer.

The output data of this program is in the “FASTA” format.

## 6 TEST AND FINDINGS

The sequence alignment test uses PSA, BLAST and FASTA. Each sequence alignment algorithm performs an alignment of sequences taken from the test datasets. Each test data starts from as small as ten records and can generate up to a maximum of one thousand records. Each record contains a number of tandem repeat patterns with a maximum of one thousand “AATG” tandem repeat patterns being generated. For the three sequence alignment algorithms, they are subjected to the same datasets and individual algorithm performance (time in milli-seconds) for each dataset recorded in a spreadsheet. Fig. (**18**) shows the results obtained for the experiment.

From the chart, it has been observed that as the number of records increases, PSA sequence alignment algorithm becomes prohibitively time-consuming. However, BLAST and FASTA were efficient for searching small and large data sets. The conclusion is that PSA is more suitable for aligning a small number of sequences. From this experiment, it is clear that PSA does not scale well with large data sets whereas BLAST and FASTA are designed to handle sequence alignments on large data sets efficiently [7].

An additional test is conducted to verify the correctness of PSA algorithm in terms of sequence alignment and compared to BLAST and FASTA algorithms. Two sequences were used as benchmark for the comparison test.

Query Sequence:


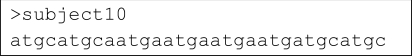


Target Sequence:


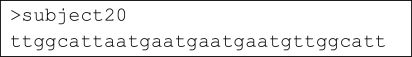


The result obtained from the test, using the query sequence and the target sequence, indicates that PSA performs the alignment correctly when benchmarked against BLAST and FASTA. The sequence alignment results from the three algorithms indicating the correct alignment, which starts from positions 9 – 24 as presented in Figs. (**19-21**).

Fig. (**22**) depicts the time taken for searching the exact tandem repeats within a given test sequence. A dataset that contains a number of sequences with different sequence lengths was used for the test. As the length of the sequence increases, the time taken to search the exact tandem repeats within a sequence increases exponentially. This is as expected due to the number of tandem repeats getting larger with longer sequence length as it would take a longer time to process all the possible ETRs with a sequence.

The following test has been conducted to search through the different sets of sequences (within a dataset) to locate a possible match for the given TR pattern. For example, “AATG” is the TR pattern and the number of TRs within the query sequence is known. The test is to search through the databases (which contain many sequences) given the query sequence detail to find a possible match. Fig. (**23**) depicts the results obtained.

From the chart, the time taken to complete an exhaustive search is proportional to the number of sequences available for search. This is as expected as more time is needed to iterate through all the sequences within datasets. During the test, correct matches were identified for all the query sequences.

Next, two identical sequences are used to test the Dotplot algorithm.

Sequence 1: AATGAATGAATGAATGAATGAATGAATGAATG

Fig. (**24**) is the result generated by the Dotplot program. An “=” sign indicates that there is an alignment. The diagonal lines formed by the “=” sign represent the alignment of the sequences.

## 7 LIMITATIONS AND DIFFICULTIES OF PRESENT WORK

The test results have indicated that the system developed needs further enhancement such as optimization of the system performance and efficiency. Some of the suggestions are:

Sequence 2: AATGAATGAATGAATGAATGAATG

- Enhancement to the existing tools, by adding more options and controls to each tool.
- Develop robust distributed software architecture to better manage the computation loads of the tools. For instance, adding a load balancer to ensure that work is evenly distributed among processors.
- As all subsequent analysis done assumes that the alignment is made without error, more focus should be placed on correctly aligning the sequences to avoid a false-positive or in this case a false arrest. Although this will diminish the speed, there is a sacrifice of speed versus accuracy that should be mentioned. An alternative approach is to take the query sequence and Blast or other similar algorithm to find candidate sequences from the larger database. Then a separate alignment algorithm that is more accurate would be implemented to increase the probability of a correct match followed by the output of possible matches with the confidence level given. Finally, TR and ATR and ETR analysis can be done.
- Fault tolerance. At this stage, the tools are not able to cope with any faults (e.g. hardware failure) during runtime; in this event, the software hangs. It would be desirable to enhance the robustness of the system for real operation needs.
- Adding more analysis tools to the existing system to provide a comprehensive software environment for sequence analysis.
- Enhancement to the system results visualisation. Current implementation is rather rudimentary as the work focus is on the overall software architecture and integration.
- As BLAST and FASTA are techniques for relating homologies, which are useful for finding relationships between sequences that are less identical. Thus one should look at EXACT match, to avoid identifying an innocent person as a criminal.

Finding of similarities using BLAST and FASTA is commonly used, one should not restrict to only for NOT identical match. These algorithms come with a confidence level between 0-100%. Unless a 100% level is obtained, one does not normally convict a person using only 85-95% confidence level. Having said that, bear in mind that DNA evidence is circumstantial which means that it can strongly suggest something but does not prove it. In all, one does not convict a person based on DNA evidence since current law does not support this method of conviction either.

## 8 CONCLUSION

The results obtained from the tests have shown that the integrated software tool performs as expected. The work suggested that by integrating BLAST and FASTA (two widely used and freely available algorithms), plus an additional implementation of PSA and TR analysis tools, with the rest of the supporting tools (database management) developed, it is entirely possible to have an initial working version of the software tool for criminal DNA analysis and detection work. The system has great potential and that the results obtained during the tests were satisfactory. The following observations can be made:

- PSA algorithm is suitable for smaller sequence alignments as compared to BLAST and FASTA, which are designed for large sequence database searches.
- All three-sequence alignment algorithms produced the same results using the given set of test data. This verified the correctness of the algorithms.
- PSA is capable of running in multi-threading mode. This may help to improve its performance if the algorithm is executed under multiple processors.
- The TR search tool has produced promising results for TR analysis within a sequence (to identify all possible TRs) and found matches against multiple TRs databases. However, the limiting factor of the tool is its performance using large data sets.
- The Dotplot works well using the test data. It is a good tool for performing preliminary sequence alignments, helping to locate segments of sequence similarities. Subsequently, these segments can be further examined using PSA, BLAST or FASTA.
- The administration tools provide user-friendly files and databases manipulation capabilities, which quicken the process of data management and persistent storage.

## Figures and Tables

**Fig. (1):**
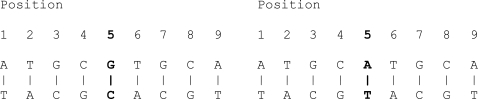
An example of sequence polymorphisms.

**Fig. (2):**
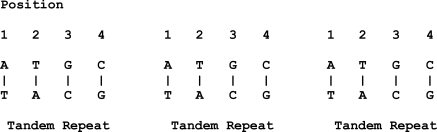
An example of length polymorphisms.

**Fig. (3):**
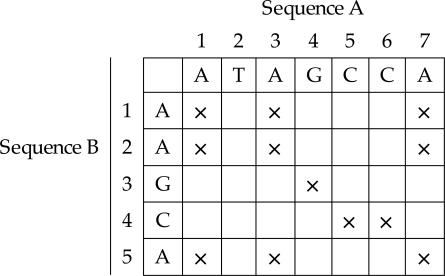
Dot Plot.

**Fig. (4):**
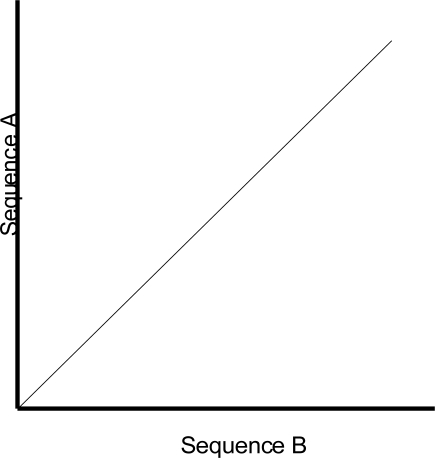
Comparison of two identical sequences.

**Fig. (5):**
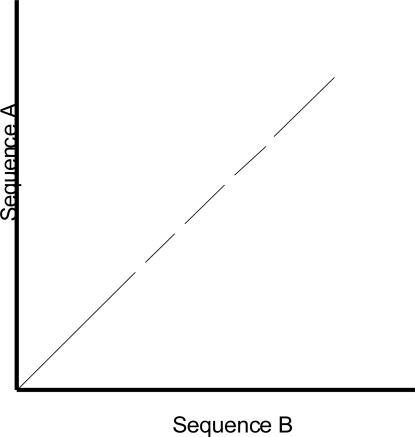
Comparison of two highly similar sequences.

**Fig. (6):**
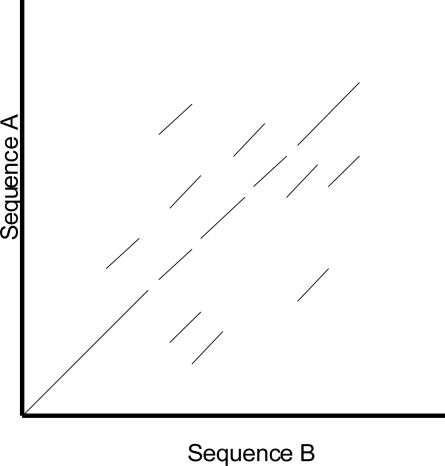
Comparison of two distantly related sequences.

**Fig. (7):**
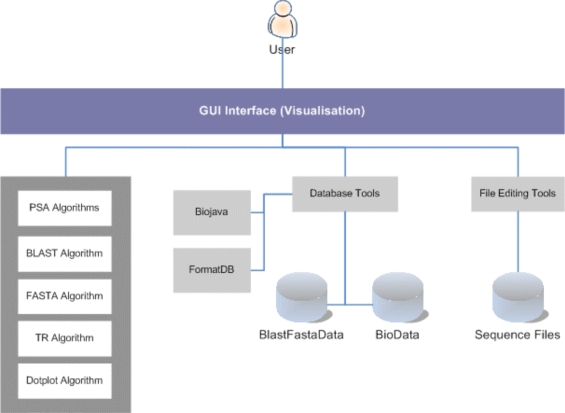
System Architecture.

**Fig. (8):**
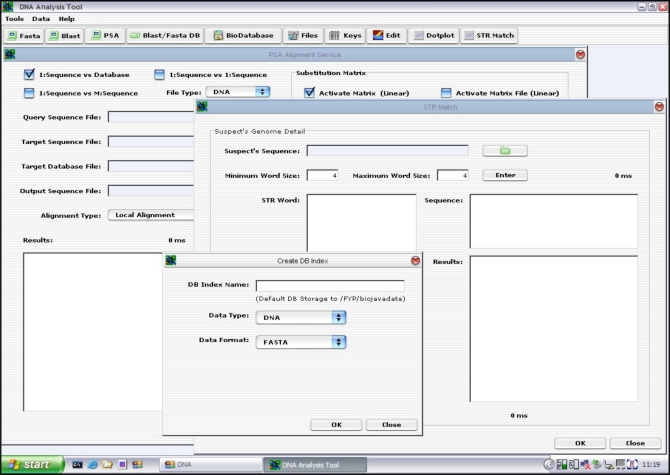
GUIMain Java Frame vs Java Internal Frame.

**Fig. (9):**
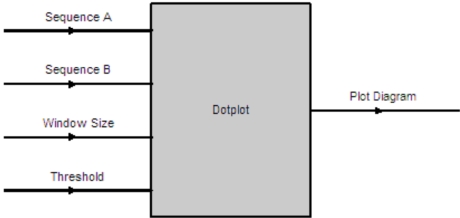
Dotplot Input & Output Parameters.

**Fig. (10):**
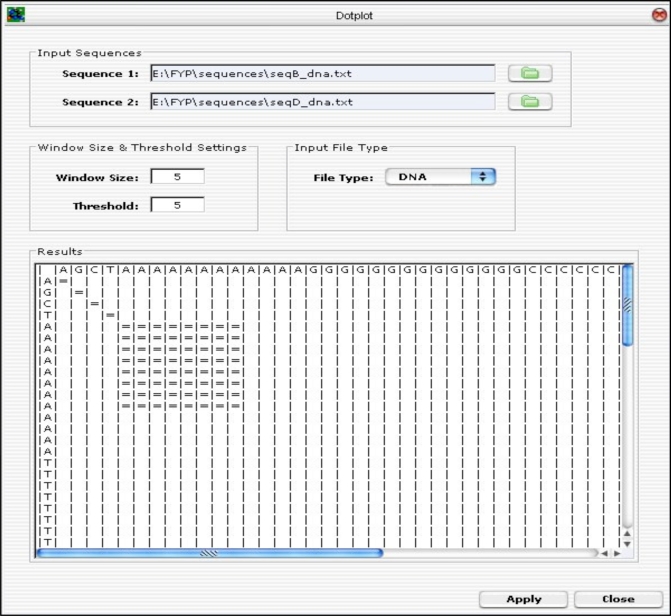
Dotplot GUI Layout.

**Fig. (12):**
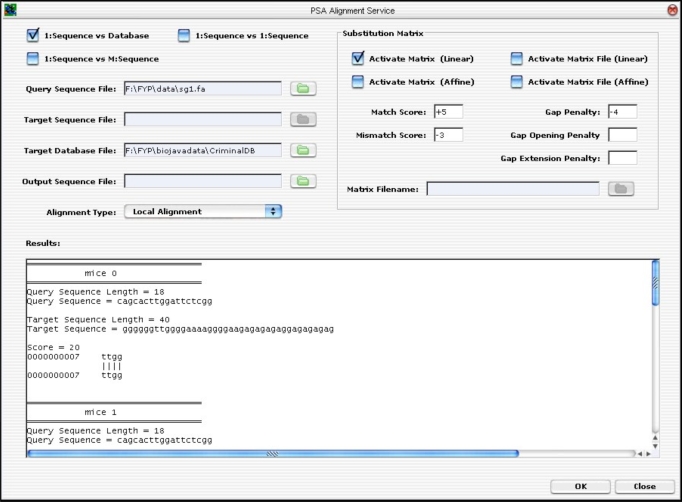
The PSA GUI Implementation.

**Fig. (13):**
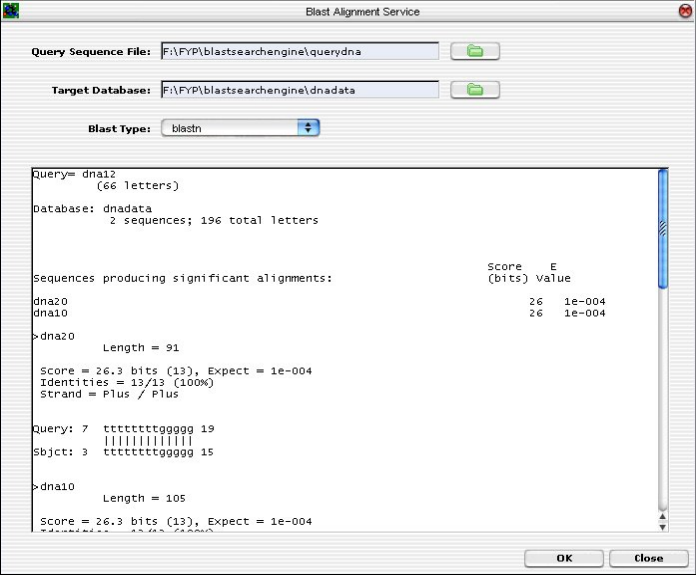
BLAST GUI Layout.

**Fig. (14):**
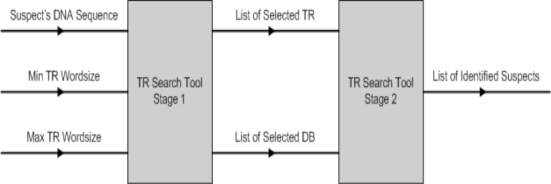
TR search tool parameters.

**Fig. (15):**
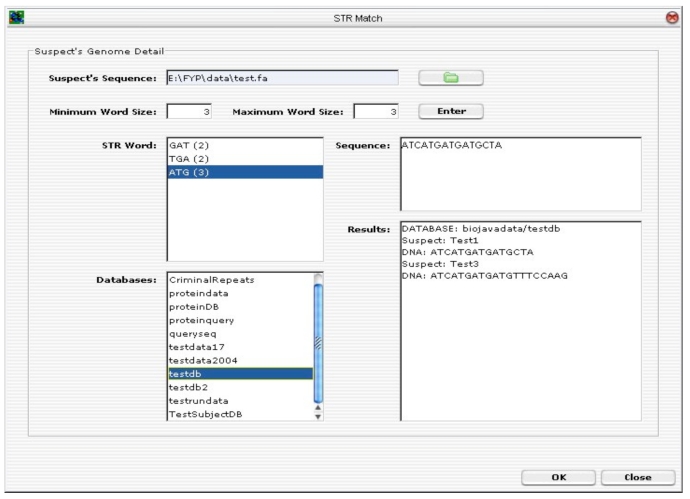
Tandem Repeats Search Tool.

**Fig. (16):**
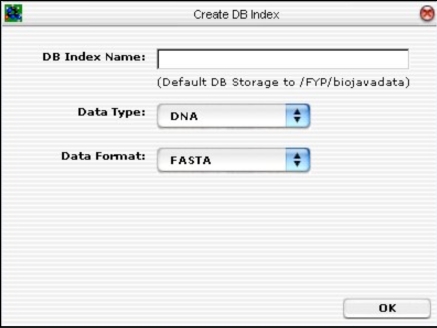
Database tool for creating BioJava compatible database.

**Fig. (17):**
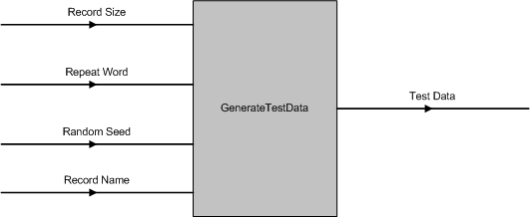
GenerateTestData() Input & Output Parameters.

**Fig. (18):**
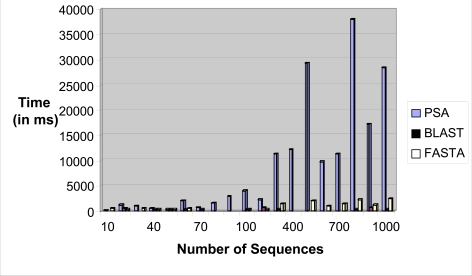
Time performance vs number of sequences bar chart.

**Fig. (19):**
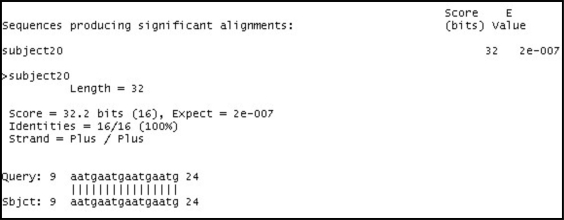
BLAST Alignment results.

**Fig. (20):**
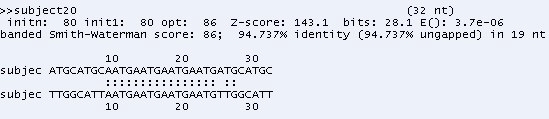
FASTA Alignment results.

**Fig. (21):**
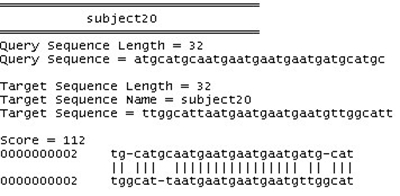
PSA Alignment results.

**Fig. (22):**
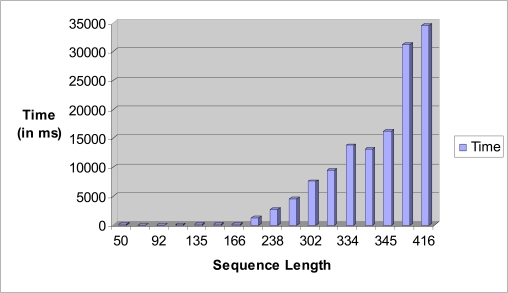
Sequence length vs time in ms bar chart for STR Analysis.

**Fig. (23):**
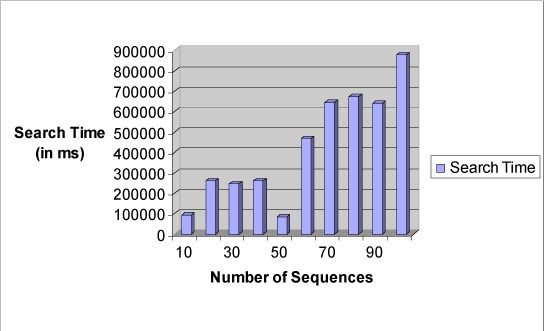
Number of sequences vs search time in ms for STR Database search.

**Fig. (24):**
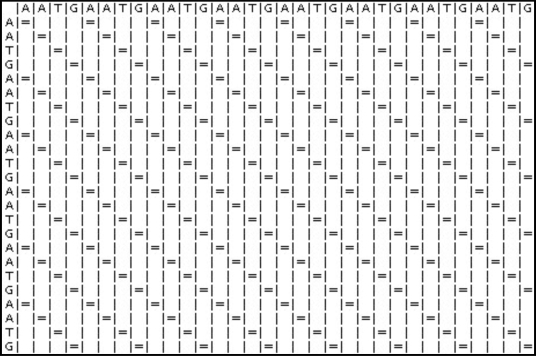
Dotplot Results.

**Table 1:** Pairwise Sequence Alignment Generalisation

Input	Two sequences *X_m_* and *Y_n_* where *m* and *n* are the length of sequences *X* and *Y* respectively. A scoring function, s( *x*, *y*), where $x\in X_{m}$ and $y\in Y_{n}$ Score for character similarity (i.e. *x* = *y*) Score for character dissimilarity (i.e. *x* ≠ *y*) Score for gap, *g* (i.e. inserting a ‘-‘ character) Example: s(*x*, *y*) = +1 if *x* = *y*, gives a score of +1 if the two character are the same s(*x*, *y*) = -1 if *x* ≠ *y*, gives a score of -1 if the two character are not the same *g* = -2, gives a score of -2 if a gap is introduced in the sequence
Task	Traverse along the two sequences and attempt to find the optimal alignment between the sequences such that the total score is maximized. During the process, gaps may be introduced to assist sequence alignment.
Output	The alignment of the two sequences with the best score. Example on DNA sequence: 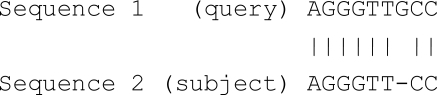 Example on Protein sequence: 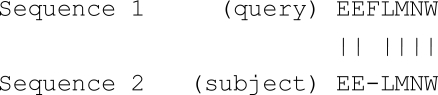

**Table 2.** Global Alignment with Linear Gap Penalty Algorithm

Input:	Given sequences of *S*_1_ and *S*_2_ with length *n* and *m* respectively. Let *s* be the scoring scheme and *g* be the linear gap penalty.
Objective:	To find the best match between sequences S_1_ and S_2_ of from one end to another.
Step 1:	Compute the similarity score of the optimal global alignment with linear gap penalty. First, construct a (*m*+1) × (*n*+1) matrix *M* and then initialise the matrix *M* with the following conditions, *M* (0, 0) =0 *M* (*i*, 0)= *i* × *g* *M* (0 , *j*)= *j* × *g* *i* and *j* are the row and column index of matrix *M* respectively and *g* is the gap penalty from the scoring scheme. Lastly, construct the rest of the remaining cells according to the recurrence relation for global alignment with linear gap penalty: $M\left(i,j\right)=\text{max}\left\{\begin{array}{c} M\left(i-1,j-1\right)+s\left(S_{1}\left[i\right],S_{2}\left[j\right]\right)\\ M\left(i-1,j\right)+g\\ M\left(i,j-1\right)+g \end{array}\right.$
Step 2:	In step 1, only the similarity score is computed. To find the alignment itself, we must find the path of choices that lead to this score. This is known as the traceback stage. In each cell, it saves pointer(s) to parent cell(s) that gives the optimal score (where the optimal score originated) *M* (*i*, *j*) contains the optimal score and the formula is as follows: *M*(*i*, *j*)= max {*M*(*i*-1, *j*-1) + *s* (*S*_1_[*i*], *S*_2_[*j*]), *M*(*i*- 1, *j*)+ *g*, *M*(*i*, *j* - 1)+ *g*}. Initialise *P* to an empty set, $P\left(i,j\right)=\emptyset$ . From the matrix top to bottom, left to right, perform the following operations to locate the traceback path for each cell. Case 1 IF (*M*(*i*, *j*) == *M*(*i* - 1, *j* - 1) + *s*(*S*_1_[*i*], *S*_2_[*j*])) then $P\left(i,j\right)\cup \left(↖\right)$ Case 2 IF (*M*(*i*, *j*)== *M*(*i* - 1, *j*) + *g*) then *P*(*i*, *j*) = $P\left(i,j\right)\cup \left(↑\right)$ Case 3 IF (*M*(*i*, *j*) == *M*(*i*, *j* -1) + *g*) then *P*(*i*, *j*) = $P\left(i,j\right)\cup \left(\leftarrow \right)$
Step 3:	Start from the final cell *P*(*m*, *n*) and follow any path back to *P* (0, 0). It is possible to obtain multiple alignments if there are more than one path leading to *P* (0, 0).
	Time complexity: O(*m*× *n*) Space complexity: O(*m* × *n*)

**Table 3.** Semi-Global Alignment with Linear Gap Penalty

Input:	Given a sequence *S*_1_ of length *m* and a sequence *S*_2_ of length *n* and a scoring scheme *S* Linear gap penalty function: *w* (*k*) = *g* × *k*, where *w*(*k*) indicates cost of a gap of length *k* and *g* is a constant.
Objective:	To find the best match between subsequence of *S*_1_ and *S*_2_. This semi-global alignment is particularly useful when lengths of sequences differ significantly and we want to align the shorter sequence in the interior of the other or to align the suffix of one sequence to the prefix of the other.
Step 1:	Compute the similarity score of the optimal semi-global alignment with linear gap penalty. First, construct a (*m*+1)×(*n*+1) matrix *M*, and then initialise the matrix *M* with the following conditions, *M* (0, 0)=0 *M* (*i*, 0)=0 *M* (0, *j*)=0 *i* and *j* are the row and column index of matrix *M* respectively. Take note of the differences. The first row and first column of the matrix *M* are initialised to zeros. Lastly, construct the rest of the remaining cells according to the recurrence relation for global alignment with linear gap penalty: $M\left(i,j\right)=\text{max}\left\{\begin{array}{c} M\left(i-1,j-1\right)+s\left(S_{1}\left[i\right],S_{2}\left[j\right]\right)\\ M\left(i-1,j\right)+g\\ M\left(i,j-1\right)+g \end{array}\right.$
Step 2:	This step is similar to the global alignment (section 2.4.1).
Step 3:	Start from the last row or the last column of matrix *M* with the maximum value. Trace until the cell (0, 0) is reached.
	Time complexity: O(*m* × *n*) Space complexity: O(m×n)

**Table 4.** Local Alignment with Linear Gap Penalty

Input:	Given sequences of *S*_1_ and *S*_2_ with length *n* and *m* respectively. Let *s* be the scoring scheme and *g* be the linear gap penalty.
Objective:	To find the best match between subsequence of *S*_1_ and *S*_2_
Step 1:	The step 1 is similar to that of semi-global alignment except we are computing the similarity score of the optimal local alignment with linear gap penalty. Take note here that there is an additional of a zero in the modified recurrence relation.
Step 2:	This step is similar to the global alignment (section 2.4.1).
Step 3:	Find the maximum value of *M* [*i*, *j*] , which can be anywhere in the matrix. Traceback pointers from the maximum cell until a cell with value 0 is reached.
	Time complexity: O(*m*×*n*) Space complexity: O(*m* × *n*)

**Table 5.** Global Alignment with Affine Gap Penalty

Input:	Given sequences of *S_1_* and *S_2_* with length *n* and *m* respectively. Let *s* be the scoring scheme, *h* be the gap opening penalty and *g* be the gap extension penalty.
Step 1:	Global alignment dynamic programming algorithm for the affine gap penalty case uses three matrices instead of one. $\left(↖\right)$*M* (*i*, *j*) = score of the best global alignment of *S*_1_[1..*i*] and *S*_2_[1..*j*] that ends in *S*_1_[*i*] matched with *S*_2_[*j*]. $\left(\leftarrow \right)$*E* (*i*, *j*) = score of the best global alignment of *S*_1_ [1..*i*] and *S*_2_ [1..*j*] that ends in a gap matched with *S*_2_ [*j*]. $\left(↑\right)$*F*(*i*, *j*) = score of the best global alignment of *S*_1_ [1..*i*] and *S*_2_ [1..*j*] that ends in *S*_1_ [*i*] matched with a gap. Construct these matrices, initialise the first row and first column of the three matrices with the following initial conditions, *M*(0,0) = 0; *M*(*i*, 0) = -∞ ; *M *(0 *, j*) = -∞ *E*(0,0) = -∞ *E*(*i*, 0)= -∞ *E*(0, *j*) = *h*+*j*×*g* *F*(0,0)= *F*(*i*, 0) = *h*+*i*×*g*; *F*(0, *j*)=-∞ Lastly, construct the rest of the remaining cells according to the recurrence relation for global alignment with affine gap penalty, $M\left(i,j\right)=s\left(S_{1}\left[i\right],S_{2}\left[j\right]\right)+\text{max}\left\{\begin{array}{c} M\left(i-1,j-1\right)\\ E\left(i-1,j-1\right)\\ F\left(i-1,j-1\right) \end{array}\right.$ $E\left(i,j\right)=\text{max}\left\{\begin{array}{c} h+g+M\left(i,j-1\right)\\ g+E\left(i,j-1\right)\\ h+g+F\left(i,j-1\right) \end{array}\right.$ $F\left(i,j\right)=\text{max}\left\{\begin{array}{c} h+g+M\left(i-1,j\right)\\ h+g+E\left(i-1,j\right)\\ g+F\left(i-1,j\right) \end{array}\right.$
Step 2:	This is the traceback stage. Fill in the rest of the three matrices from the top to the bottom, and from the left to the right and then store the corresponding pointers to the parent cells in each matrix.
Step 3:	Start from the cell with the largest value of either *M*(*m*, *n*), *E*(*m*, *n*) *or F*(*m*, *n*). Trace back pointers until the cell *M*(0,0) is reached.

**Table 6.** Semi-Global Alignment with affine gap penalty

Input:	Given sequences of *S*_1_ and *S*_2_ with length *n* and *m* respectively. Let *s* be the scoring scheme, *h* be the gap opening penalty and *g* be the gap extension penalty.
Step1:	The step 1 is similar to that of global alignment (section 2.4.4) except that the semi-global alignment dynamic programming algorithm for the affine gap penalty with three matrices is used here. Also, one of the initial conditions is *F*(0, 0) = -∞ ; *F*(*i*, 0) = -∞ *F*(0, *j*) = -∞
Step2:	As step 2 of section 2.4.4.
Step3:	Start from the last row or last column of the matrix *M* with the largest value. Traceback pointers until the cell (0, 0) is reached.

**Table 7.** Local Alignment with Affine Gap Penalty

Input:	Given sequences of *S*_1_ and *S*_2_ with length *n* and *m* respectively. Let *s* be the scoring scheme, *h* be the gap opening penalty and *g* be the gap extension penalty.
Step1:	Step 1 is similar to the semi-global alignment (section 2.4.5) except that the local alignment dynamic programming algorithm for the affine gap penalty case with three matrices is used here. Also, there is an additional of a zero in the modified recurrence relation max(M (i,j)).
Step2:	As step 2 of section 2.4.5.
Step3:	Start from the cell with the highest value of matrix *M*. Trace back pointers until a cell with zero value of the matrix *M* is reached.

**Table 8.** Tandem Repeats Search Algorithms

Method	Characteristic	Key Steps
Tandem Repeat Search via data compression approach [Rival 1997]	Works well for short tandem patterns (less than four). Assume tandem repeat zone must begin and end with exact tandem pattern.	First, locate all the PTRs within a sequence. Then, the algorithm will attempt to compress each adjacent PTR. If there is a compression gain, then it is an ATR region. This process is repeated for each adjacent ATR. From the ATR region, derive a new compressed sequence from the original one. A function is used to evaluate the likelihood of each ATR region as a tandem repeat based on the compression gain criteria.
Tandem Repeat Finder [Benson 1999]	This method is considered to be more general as compared to other TR methods. Based on Bernoulli trials concept. Uses a sliding window of size *k* and transverses along the nucleotide sequence.	First a small window of size *k* is constructed. Then, create an exhaustive list of *k*-length strings. There should be 4^*k*^ of such strings, which are known as probes. For each *k*-length probe *p*, slide along the nucleotide sequence using the *k*-size window. Insert position *i* into the list *H_p_*, if there is a match for probe *p* at position *i*. From the list *H_p_*, scan *H_p_* for all *j* < *i*. The distance *d* = *i* – *j* is a possible tandem repeat. Keep a distance list *D_d_*. Update it every time a match at distance *d* is detected. During the analysis phase, use dynamic programming to align a potential tandem pattern of size *d* with its surrounding sequence. If two or more copies are aligned, then the pattern is reported as a tandem repeat.
